# Stagnation Point Flow and Mass Transfer with Chemical Reaction past a Stretching/Shrinking Cylinder

**DOI:** 10.1038/srep04178

**Published:** 2014-02-26

**Authors:** Najwa Najib, Norfifah Bachok, Norihan Md. Arifin, Anuar Ishak

**Affiliations:** 1 Department of Mathematics and Institute for Mathematical Research, Universiti Putra Malaysia, 43400 UPM Serdang, Selangor, Malaysia; 2 School of Mathematical Sciences, Universiti Kebangsaan Malaysia, 43600 UKM Bangi, Selangor, Malaysia

## Abstract

This paper is about the stagnation point flow and mass transfer with chemical reaction past a stretching/shrinking cylinder. The governing partial differential equations in cylindrical form are transformed into ordinary differential equations by a similarity transformation. The transformed equations are solved numerically using a shooting method. Results for the skin friction coefficient, Schmidt number, velocity profiles as well as concentration profiles are presented for different values of the governing parameters. Effects of the curvature parameter, stretching/shrinking parameter and Schmidt number on the flow and mass transfer characteristics are examined. The study indicates that dual solutions exist for the shrinking cylinder but for the stretching cylinder, the solution is unique. It is observed that the surface shear stress and the mass transfer rate at the surface increase as the curvature parameter increases.

The stagnation flow is about the fluid motion near the stagnation point. The fluid pressure, the heat transfer and the rate of mass deposition are highest in the stagnation area. Wang^1^ investigated the stagnation flow towards a shrinking sheet and found that the convective heat transfer decreases with the shrinking rate due to an increase in the boundary layer thickness. He obtained dual solutions and unique solution for specific values of the ratio of shrinking and straining rates. The similar flow over a shrinking sheet in a micropolar fluid was investigated by Ishak et al.^2^. Bachok et al.^3^ considered the stagnation-point flow and heat transfer from a warm, laminar liquid flow to a melting stretching/shrinking sheet. This problem was then extended to a micropolar fluid by Yacob et al.^4^. The effects of homogeneous-heterogeneous reactions on the steady boundary layer flow near the stagnation-point on a stretching/shrinking surface were studied by Bachok et al.^5^. Later, Bachok et al.^6^,^7^ studied the boundary layer stagnation-point flow towards a stretching/shrinking sheet in a nanofluid. Bhattacharyya^8^ discussed the existence of dual solutions in the boundary layer flow and mass transfer with chemical reaction passing through a stretching/shrinking sheet. He found that the concentration boundary layer thickness decreases with increasing values of the Schmidt number and the reaction-rate parameter for both solutions. The problem considered by Wang^1^ was extended by Bhattacharyya^9^, Bhattacharyya and Layek^10^, Bhattacharyya and Pop^11^, Bhattacharyya et al.^12^,^13^ and Lok et al.^14^ to various physical conditions.

The flow over a cylinder is considered to be two-dimensional if the body radius is large compared to the boundary layer thickness. On the other hand, for a thin or slender cylinder, the radius of the cylinder may be of the same order as that of the boundary layer thickness. Therefore, the flow may be considered as axi-symmetric instead of two-dimensional^15^,^16^. The study of steady flow in a viscous and incompressible fluid outside a vertical cylinder has been done by Ishak^17^ and Bachok and Ishak^18^. The effect of slot suction/injection as studied by Datta et al.^15^ and Kumari and Nath^16^ may be useful in the cooling of nuclear reactors during emergency shutdown, where a part of the surface can be cooled by injection of coolant (Ishak et al.^19^). Lin and Shih^20^,^21^ considered the laminar boundary layer and heat transfer along horizontally and vertically moving cylinders with constant velocity and found that the similarity solutions could not be obtained due to the curvature effect of the cylinder. Ishak and Nazar^22^ showed that the similarity solutions may be obtained by assuming that the cylinder is stretched with linear velocity in the axial direction and noted that their study is the extension of the papers by Grubka and Bobba^23^ and Ali^24^, from a stretching sheet to a stretching cylinder.

The addition of chemical reaction in the boundary layer flow has huge applications in air and water pollutions, fibrous insulation, atmospheric flows and many other chemical engineering problems. Hayat et al.^25^ discussed the mass transfer in the steady two-dimensional MHD boundary layer flow of an upper-convected Maxwell fluid past a porous shrinking sheet in the presence of chemical reaction and expressions for the velocity and concentration profiles were obtained using HAM. Bhattacharyya and Layek^26^ discussed the behavior of chemically reactive solute distribution in MHD boundary layer flow over a permeable stretching sheet, vertical stretching sheet^27^ and stagnation-point flow over a stretching sheet^28^. The aim of the present study is to extend the paper by Bhattacharyya^8^ to a cylindrical case. We investigate the skin friction and the mass transfer characteristics at the solid-fluid interface in the presence of chemical reaction. To the best of our knowledge, this problem has not been studied before and all results are new.

## Problem formulation

**Figure 1:**
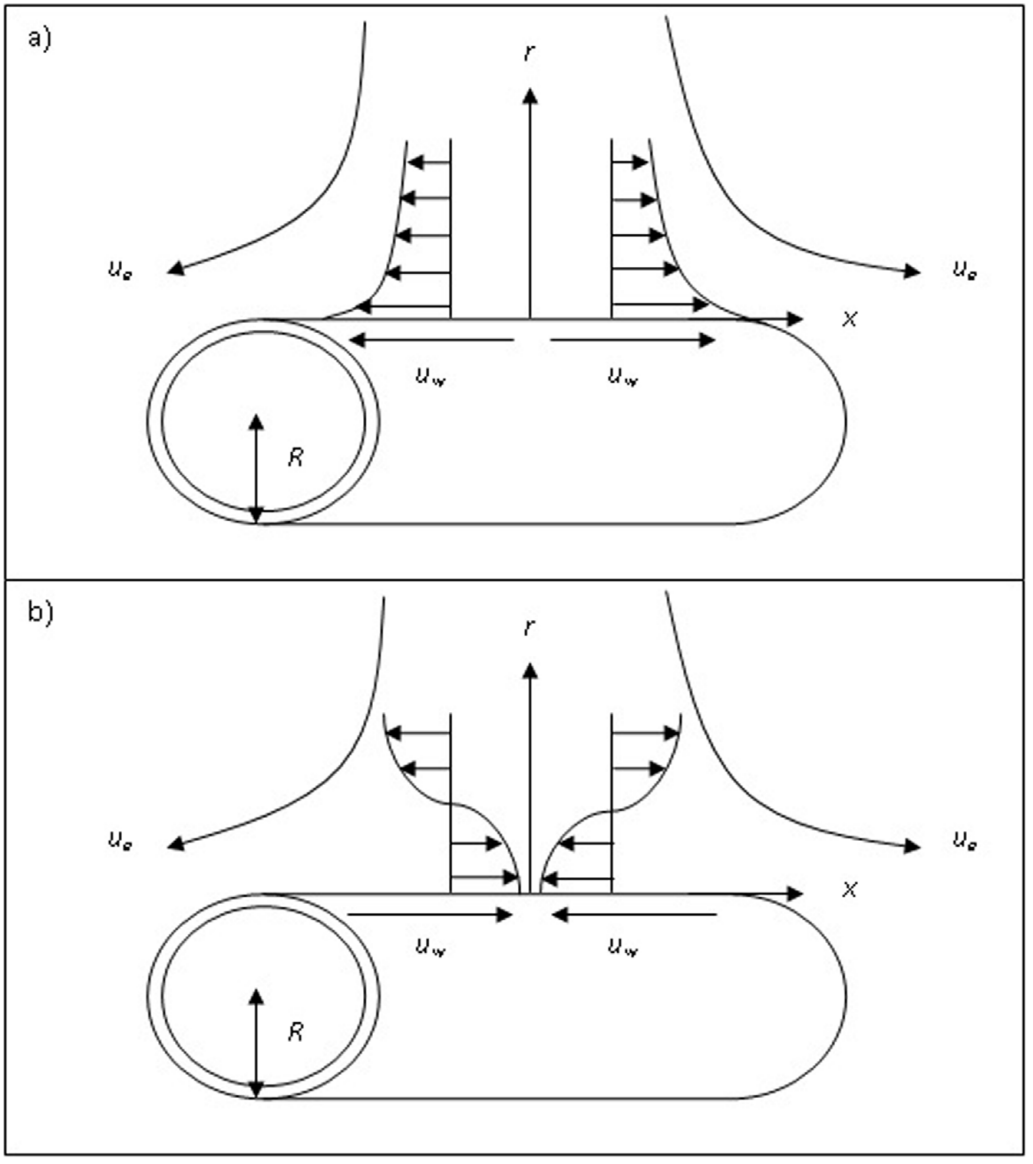
Physical model and coordinate system for cylinder (a) stretching case (b) shrinking case.

Consider a steady stagnation-point flow towards a horizontal linearly stretching/shrinking cylinder with radius *R* placed in an incompressible viscous fluid of constant temperature *T_w_* and chemically reactive species undergoing first order chemical reaction as shown in Fig. 1. It is assumed that the free stream and the stretching/shrinking velocities are *u_e_* = *a x*/*L* and *u_w_* = *c x*/*L* respectively, where *a* and *c* are constants, *x* is the coordinate measured along the cylinder and *L* is the characteristics length. The boundary layer equations are (Ishak^17^; Bhattacharyya^8^) 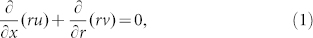


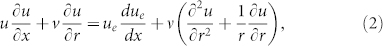


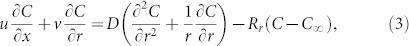
where *r* is the coordinate measured in the radial direction, and *u* and *v* are the velocity components in the *x* and *r* directions, respectively. Further, *T* is the temperature in the boundary layer, *v* is the kinematic viscosity coefficient, *C* is the concentration, *C*_∞_ is the constant concentration in the free stream, *D* is the diffusion coefficient and *R_r_* denotes the reaction rate of the solute.

The boundary conditions are 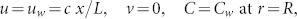


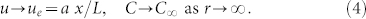
We look for similarity solutions of equations (1)–(3), subject to the boundary conditions (4), by writing 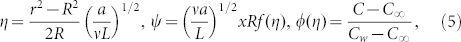
where *η* is the similarity variable, *ψ* is the stream function defined as 
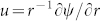
 and 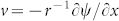, which identically satisfies equation (1). By defining *η* in this form, the boundary conditions at *r* = *R* reduce to the boundary conditions at *η* = 0, which is more convenient for numerical computations.

Substituting (5) into equations (2) and (3), we obtain the following nonlinear ordinary differential equations: 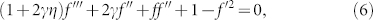


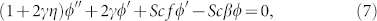
subject to the boundary conditions (4) which become 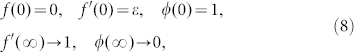
where *γ* is the curvature parameter, *Sc* is the Schmidt number, *β* is the reaction-rate parameter defined respectively as 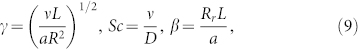
and *ε* = *c*/*a* is the stretching/shrinking parameter with *ε* > 0 is for stretching and *ε* < 0 is for shrinking.

The main physical quantities of interest are the value of *f*″(0), being a measure of the skin friction, and the concentration gradient −*ϕ*′(0). Our main aim is to find how the values of *f*″(0) and −*ϕ*′(0) vary in terms of parameters *γ*, *Sc* and *β*. When *γ* = 0 (flat plate), the present problem reduces to those considered by Bhattacharyya^8^.

## Results and Discussion

Numerical solutions to the ordinary differential equations (6) and (7) with the boundary conditions (8) form a two-point boundary value problem (BVP) and are solved using a shooting method, by converting them into an initial value problem (IVP). This method is very well described in the recent papers by Bhattacharyya^8^, Bhattacharyya et al.^12^ and Bachok et al.^29^. In this method, we choose suitable finite values of *η*, say *η*_∞_, which depend on the values of the parameters considered. First, the system of equations (6) and (7) is reduced to a first-order system (by introducing new variables) as follows: 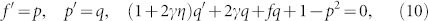


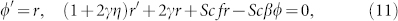
with the boundary conditions 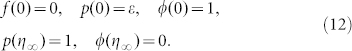
Now we have a set of ‘partial’ initial conditions 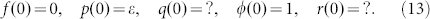
As we notice, we do not have the values of *q*(0) and *r*(0). To solve Eqs. (10) and (11) as an IVP, we need the values of *q*(0) and *r*(0), i.e., *f*″(0) and *ϕ*′(0). We guess these values and apply the Runge-Kutta-Fehlberg method in maple software, then see if this guess matches the boundary conditions at the very end. Varying the initial slopes gives rise to a set of profiles which suggest the trajectory of a projectile ‘shot’ from the initial point. That initial slope is sought which results in the trajectory ‘hitting’ the target, that is, the final value (Bailey et al.^30^).

To determine either the solution obtained is valid or not, it is necessary to check the velocity and the concentration profiles. The correct profiles must satisfy the boundary conditions at *η* = *η*_∞_ = 30 asymptotically. This procedure is repeated for other guessing values of *q*(0) and *r*(0) for the same values of parameters. If a different solution is obtained and the profiles satisfy the far field boundary conditions asymptotically but with different boundary layer thickness, then the solution is also a solution to the boundary-value problem (second solution). This method has been successfully used by the present authors to solve various problems related to the boundary layer flow (see Najib et al.^31^ and Bachok et al.^3^,^7^).

Table 1 shows the variations and comparison of *f*″(0) with those of previous researcher (Bhattacharyya^8^) for the flat-plate case, which show a good agreement, thus give confidence that the numerical results obtained are accurate. Other than that, the values of *f*″(0) for *γ* = 0.2 and *γ* = 0.4 are also included in Table 1 for future references.

**Figure 2:**
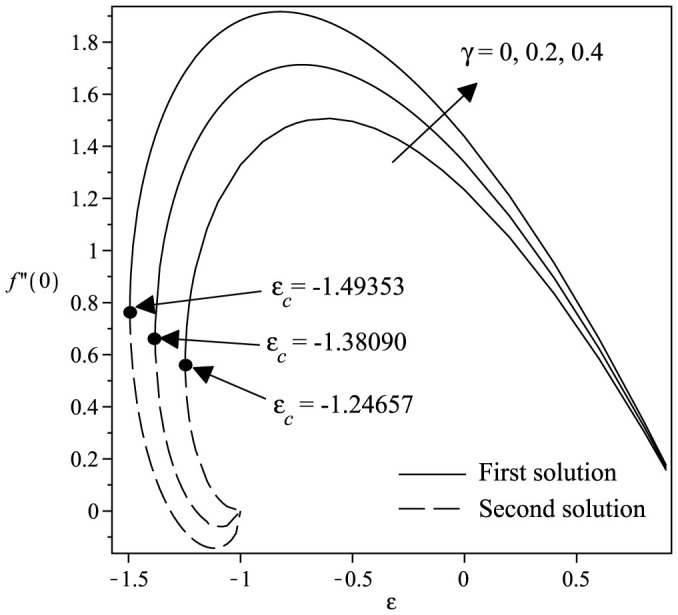
Skin friction coefficient *f*″(0) with *ε* for various values of *γ*.

**Figure 3:**
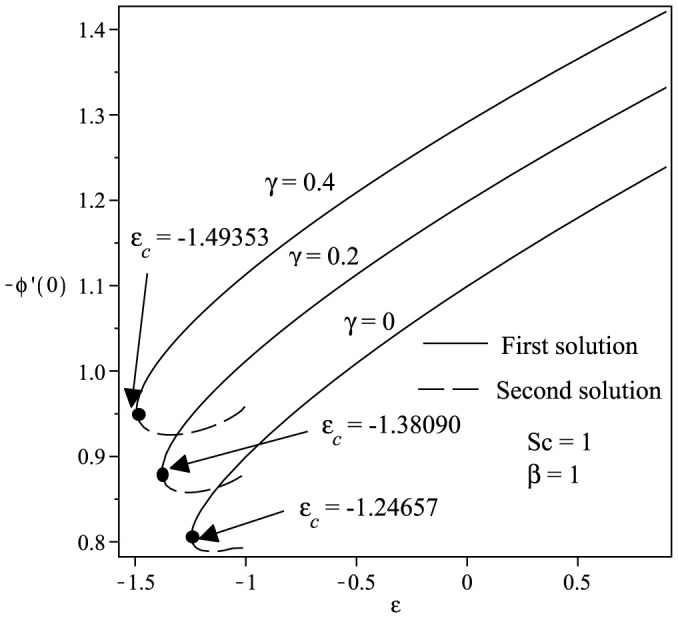
Variation of −*ϕ*′(0) with *ε* for various values of *γ* when Sc = 1 and *β* = 1.

**Figure 4:**
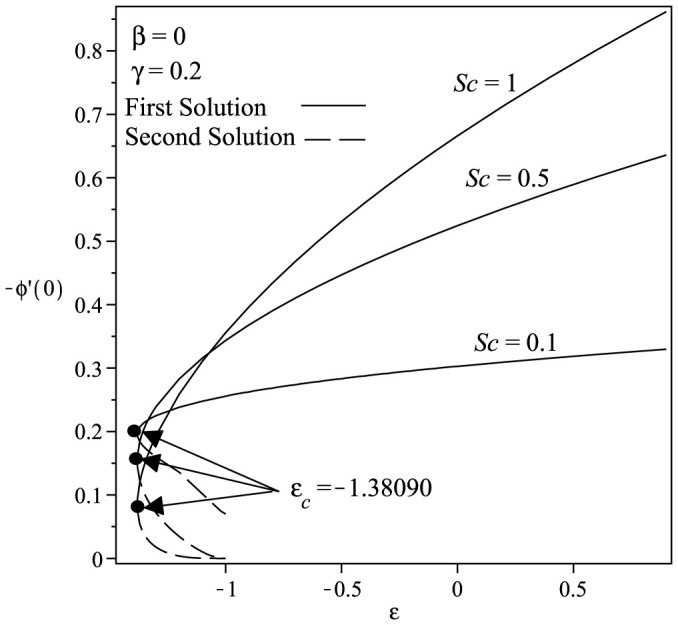
Variation of −*ϕ*′(0) with *ε* for various values of *Sc* when *β* = 0 and *γ* = 0.2.

**Figure 5:**
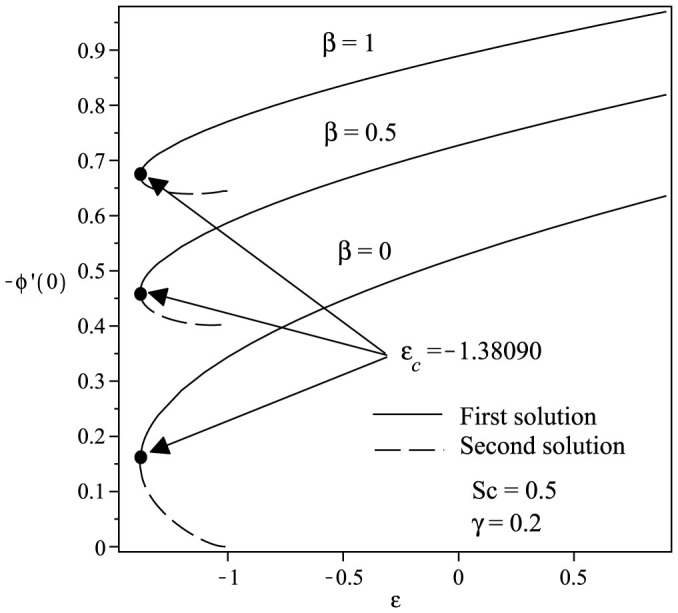
Variation of −*ϕ*′(0) with *ε* for various values of *β* when Sc = 0.5 and *γ* = 0.2.

Variations of the skin friction coefficient *f*″(0) and the concentration gradient −*ϕ*′(0) with *ε* and *γ* are shown in Figs. 2 and 3 when *Sc* = 1 and *β* = 1. From the figures, we can see that there are regions of unique solutions for *ε* ≥ −1.0, dual solutions for *ε_c_* < *ε* < −1.0 and no solutions for *ε* < *ε_c_* < −1.0. Therefore, the solutions exist up to the critical value *ε* = *ε_c_* (<−1.0). The boundary layer approximation breaks down at *ε* = *ε_c_*; thus we are unable to obtain further results for *ε* < *ε_c_*. Beyond this value, the boundary later has separated from the surface. The critical value of *ε* (say *ε_c_*) are presented in Table 2, which show a very good agreement with that of Bhattacharyya^8^, for the flat plate case (*γ* = 0). It is evident from figures 2,3,4,5 and Table 2 that 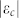 increases with an increase in the curvature parameter *γ*. The range of *ε* for which the solution exists is larger for *γ* > 0 (cylinder) compared to *γ* = 0 (flat plate). Thus, this demonstrates that a cylinder increases the range of existence of the similarity solutions to the equations (6)–(8) compared to a flat plate, i.e. the boundary layer separation is delayed for a cylinder. The results shown in Fig. 2 also indicate that as the curvature parameter *γ* increases, the skin friction coefficient *f*″(0) also increases. Fig. 3 shows the values of concentration gradient −*ϕ*′(0) which are proportional to the rate of mass transfer with an increase of *γ* when Sc = 1 and *β* = 1. Moreover, Fig. 4 presents the variation of the concentration gradient −*ϕ*′(0) for various values of Schmidt number Sc when *β* = 0 and *γ* = 0.2. It is seen that when Schmidt number Sc increases, the concentration gradient also increases. Fig. 5 shows the variation of concentration gradient −*ϕ*′(0) for increasing *β* when Sc = 0.5 and *γ* = 0.2. In general, increasing *β* is to increase the concentration gradient at the surface. The mass transfer increases with increasing values of *ε* for the first solution, but decreases with increasing *ε* for the second solution.

**Figure 6:**
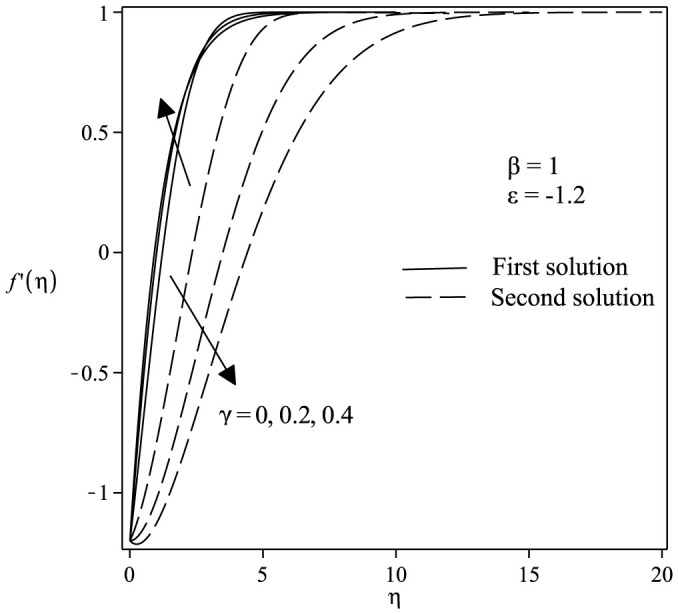
Velocity profiles *f*′(*η*) for various values of *γ* when *β* = 1, *ε* = −1.2 and *Sc* = 1.

**Figure 7:**
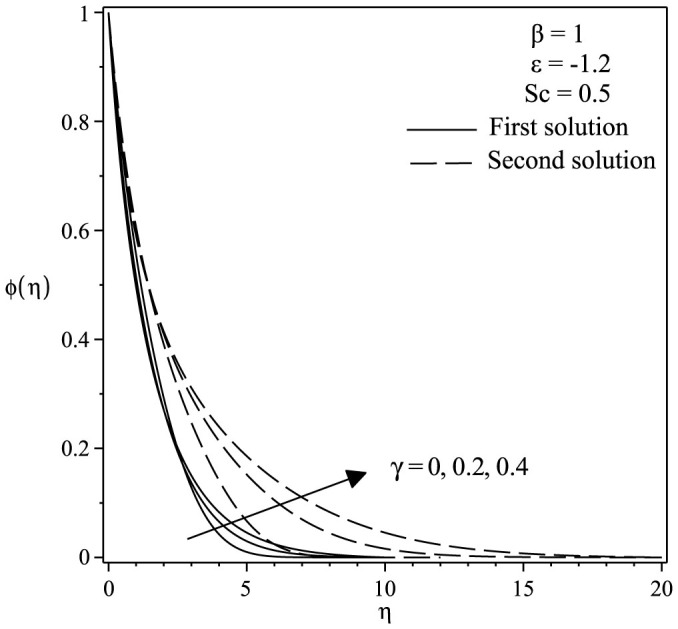
Concentration profiles *ϕ*(*η*) for various values of *γ* when *β* = 1, *ε* = −1.2 and *Sc* = 0.5.

**Figure 8:**
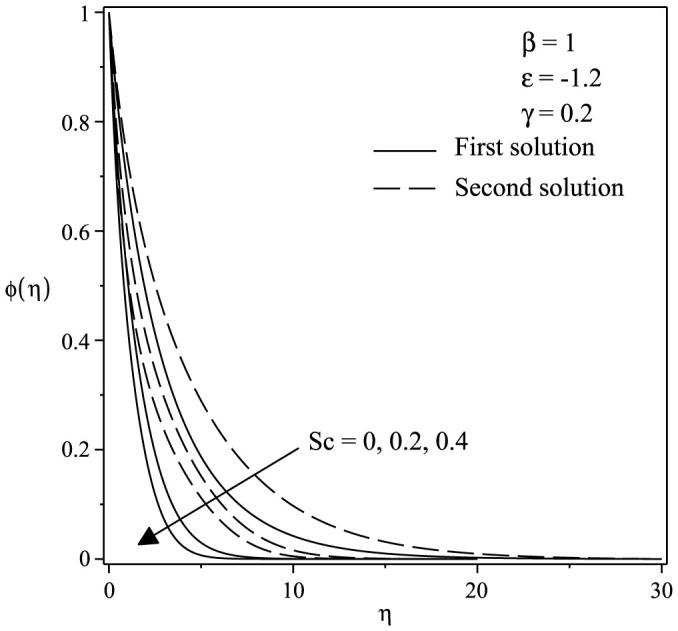
Concentration profiles *ϕ*(*η*) for various values of *Sc* when *β* = 1, *ε* = −1.2 and *γ* = 0.2.

**Figure 9:**
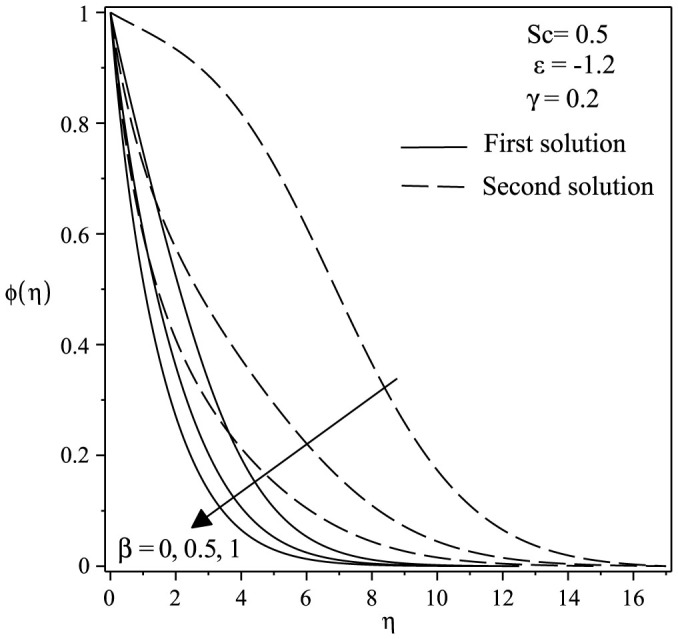
Concentration profiles *ϕ*(*η*) for various values of *β* when *ε* = −1.2, *γ* = 0.2 and *Sc* = 0.5.

**Figure 10:**
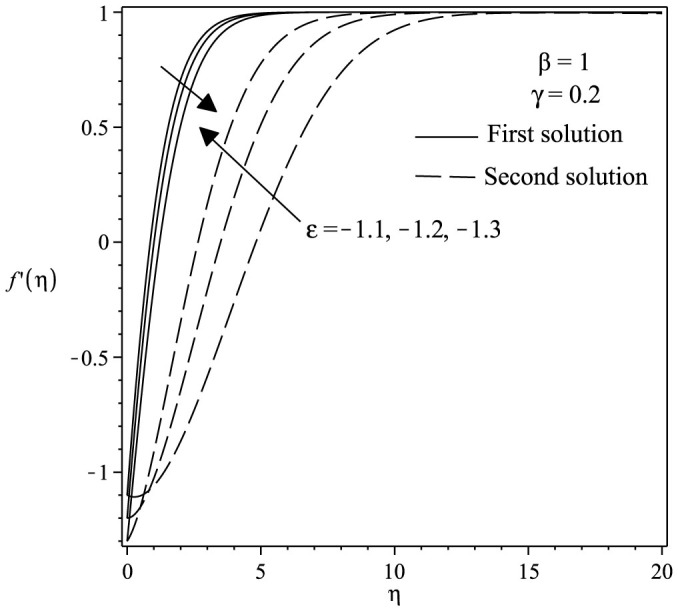
Velocity profiles *ϕ*(*η*) for various values of *ε* when *β* = 1 and *γ* = 0.2.

**Figure 11:**
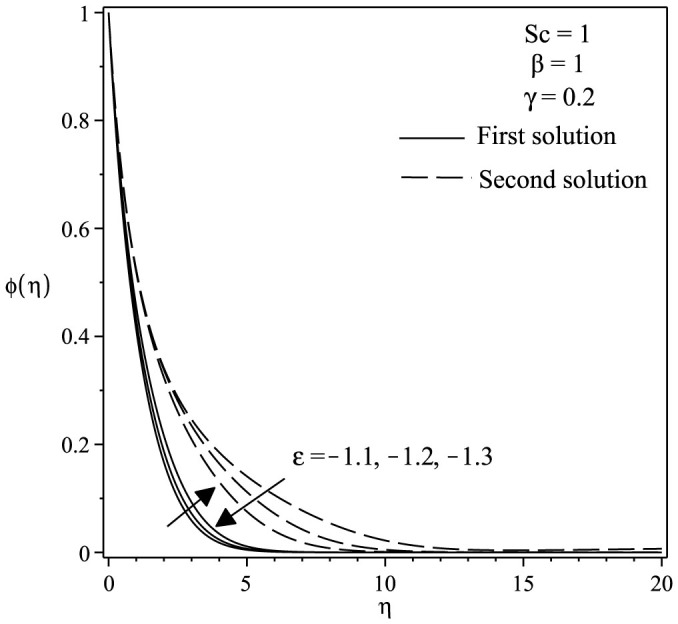
Concentration profiles *ϕ*(*η*) for various values of *ε* when *β* = 1, *γ* = 0.2 and *Sc* = 1.

The variations of velocity and concentration profiles for different values of *γ*, *ε*, Sc and *β* are display in Figs. 6,7,8,9,10,11, which show that the far-field boundary conditions are satisfied, and thus support the validity of the numerical results obtained. The dual velocity profiles *f*′(*η*) in Fig. 6 show that the velocity increases with increasing *γ* for the first solution and conversely for the second solution, it decreases. It is to be noted that the momentum boundary layer thickness for the second solution is thicker than the thickness of the first solution. On the other hand, it should be mentioned that the first solutions are stable and physically realizable, while the second solutions are not. The procedure for showing this has been described by Weidman et al.^32^, Merkin^33^, and recently by Postelnicu and Pop^34^, so that we will not repeat it here. From Fig. 7, we noticed that the value of concentration profile *ϕ*(*η*) initially decreases with *γ* and after that for large *η*, changing the nature it increases with *γ*. In Fig. 8, the effects of Schmidt number Sc on the concentration profile *ϕ*(*η*) is exhibited. The dual concentration profiles of Fig. 8 demonstrate that concentration decreases with Sc. As a result, the concentration boundary layer thickness reduces with enhancement of Sc. Fig. 9 presents the concentration profile *ϕ*(*η*) at a fixed value of Sc, *ε* and *γ* for both solutions, which shows that the boundary layer thickness decreases with increasing *β*.

## Conclusion

We have theoretically investigated how the governing parameters, i.e. curvature parameter *γ*, stretching/shrinking parameter *ε* and Schmidt number Sc, influence the boundary layer flow and mass transfer with chemical reaction characteristics on a stretching/shrinking cylinder. It was found that the solutions for the stretching case are unique but for the shrinking case, there are dual solutions for a certain range of the stretching/shrinking parameter. The curvature parameter *γ* increases the range of existence of the similarity solutions, which in turn delays the boundary layer breakdown. Hence, the boundary layer separation is delayed for a cylinder (*γ* > 0) compared to a flat plate (*γ* = 0). Further, it was found that increasing the curvature parameter *γ* is to increase the surface shear stress and the mass transfer at the surface. The concentration boundary layer thickness decreases with increasing values of Schmidt number and reaction-rate parameter for both solutions.

## Author Contributions

N.N. and N.B. conceived and designed the research. N.N. performed the results. N.N. and N.B. analyzed the results. N.N., N.B., N.M.A. and A.I. contributed to the interpretation of the results. N.N., N.B. and A.I. wrote the manuscript, N.B. wrote the Methods, while N.N., N.B., N.M.A. and A.I. contributed to the revisions.

## Figures and Tables

**Table 1:** Comparison of the values of skin friction coefficient *f*″(0) for several values of *ε* and *γ*

	Bhattacharyya^8^		Present study	
ε	γ = 0	γ = 0	γ = 0.2	γ = 0.4
−0.25	1.4022405	1.4022408	1.5396153	1.6672783
−0.50	1.4956697	1.4956696	1.6705695	1.8307527
−0.75	1.4892981	1.4892983	1.7125346	1.9119385
−1.00	1.3288169[0]	1.3288169[0]	1.6297678[0]	1.8836199[0]
−1.15	1.0822316[0.1167023]	1.0822316[0.1167022]	1.4850052[−0.0401474]	1.7911552[−0.1382962]
−1.20	0.9324728[0.2336491]	0.9324736[0.2336491]	1.4106126[0.0015206]	1.7432654[−0.1165759]
−1.2465	0.5842915[0.5542856]	0.5842915[0.5542856]	1.3231819[0.0633109]	1.6863469[−0.0802947]
−1.24657	0.5745268[0.5639987]	0.5745347[0.5639987]	1.3230334[0.0634236]	1.6862537[−0.0803612]
−1.30	-	-	1.1888761[0.1723510]	1.6059229[−0.0180635]
−1.40	-	-	-	1.3838578[0.1797279]

[ ] second solution.

**Table 2:** Variations of *ε_c_* with curvature parameter *γ*

γ	Bhattacharyya^8^	Present study
0	−1.24657	−1.24657
0.2		−1.38090
0.4		−1.49353
